# Early oxygen therapy does not protect the brain from vasogenic edema following acute ischemic stroke in adult male rats

**DOI:** 10.1038/s41598-017-02748-3

**Published:** 2017-06-12

**Authors:** Elmira Pasban, Hamdollah Panahpour, Akbar Vahdati

**Affiliations:** 1 Department of Biology, Fars Science and Research Branch, Islamic Azad University, Fars, Iran; 2 Department of Biology, Shiraz Branch, Islamic Azad University, Shiraz, Iran; 3 Department of Physiology, Medical School, Ardabil University of Medical Sciences, Ardabil, Iran

## Abstract

Brain edema aggravates primary brain injury and increases its mortality rate after ischemic stroke. It is believed that normobaric oxygen therapy (NBO) may produce neuroprotective effects against ischemic stroke; however, reports have been controversial, and its effects on vasogenic brain edema as a major complication of brain ischemia have not been clarified. The present study investigates the effects of NBO on cerebral edema and blood – brain barrier integrity using rat model of ischemic stroke. Transient focal cerebral ischemia was induced in adult male Sprague-Dawley rats by left middle cerebral artery occlusion (MCAO) for 90 min followed by 24 h reperfusion. Early NBO supplementation was started 15 min after MCAO and continued for 90 min. The results of the present study show that early oxygen therapy following acute ischemic stroke does not reduce vasogenic brain edema, nor does it protect against oxidative stress-induced BBB destruction. Additionally, cerebral edema formation occurs in conjunction with an increased mortality rate, serious brain injury, and impairment of brain antioxidant power. These findings suggest that further experimental studies should be carried out to clarify the beneficial effects and potential side effects of early oxygen therapy in acute ischemic stroke before its clinical use.

## Introduction

Brain edema is a life-threatening complication of cerebral infarction, which aggravates ischemic brain injury by compression of the cerebral vasculature and reduction of blood flow to the penumbral region^1^. Vasogenic edema is characterized by increased extracellular fluid volume due to destruction of the blood-brain barrier (BBB)^2^. This type of edema develops within the first few hours to days after a stroke. Transient brain ischemia and subsequent reperfusion enhances the production of oxygen free radicals and lipid peroxidation as one of the main causes of BBB destruction and subsequent vasogenic cerebral edema^3, 4^.

Tissue hypoxia is the main cause of cell death following cerebral ischemia. Hence, it is believed that brain tissue oxygenation might be a logical and effective strategy for ischemic stroke treatment. Previous studies have suggested a neuroprotective effect of normobaric oxygen therapy (NBO) when used for a short time and early after the occurrence of ischemic stroke^5–7^. However, there are also controversial reports that NBO does not produce neuroprotective effects^8^ or that exacerbates brain injury^9^. It was reported that oxygen therapy in stroke patients may increase mortality rates due to excessive production of reactive oxygen species (ROS) and subsequent lipid peroxidation^10–12^. In addition, it was suggested that oxygen therapy may impair cerebral blood flow by vasoconstriction and worsen stroke outcome^12, 13^.

Due to the profound controversy surrounding oxygen therapy in acute ischemic stroke and limited availability of data about its effects on brain edema as a major complication of brain ischemia, in this study, we evaluated the effects of short and early oxygen therapy on ischemic brain injury and behavioral outcome at the first step. In the second step, we assessed the effects of oxygen therapy on vasogenic brain edema, and finally, we quantified BBB damage as a result of lipid peroxidation and brain antioxidant capacity with FRAP assay.

## Methods

### Animal model and experimental protocol

The institutional animal ethics committee of Ardabil University of Medical Sciences, which follows the National Institutes of Health guidelines for the care and use of animals, approved all protocols and experiments in this study (Ethic code No IR.ARUMS.REC.1394.17). Male adult Sprague-Dawley rats (Pasteur institute of Iran, Karaj, Iran) weighing 370–400 g and 22–24 weeks old were randomly allocated to three main groups:

*Group 1* (sham, n = 32), rats were subjected to surgery of the neck region without being exposed to ischemia. Animals spontaneously breathed room air during the experimental period.

*Group 2* (control ischemic, n = 36), rats experienced brain ischemia by 90 min middle cerebral artery occlusion (MCAO) followed by 24 h reperfusion and spontaneously breathed room air.

*Group 3* (NBO treated ischemic rats, n = 36), rats experienced ischemia and reperfusion as in group 2 and received early NBO (95% O2 +5% CO_2_, 3 L/min) using an anesthesia box^14, 15^. Normobaric oxygen therapy was started 15 min after MCAO and continued for 90 min.

Four subgroups of animals were randomly studied within each main group. The first subgroup was evaluated for sensorimotor deficits and infarct size. Detection of brain edema formation was performed in the second subgroup. The third subgroup was assessed for BBB permeability. Assessment of brain tissue antioxidant power was carried out in the fourth subgroup. Animals that died during reperfusion were excluded from the study, and the mortality rate was calculated as an important index for investigation of the NBO treatment.

### Induction of transient focal cerebral ischemia

Animals were anesthetized by injection of chloral hydrate (400 mg/kg, IP) and subjected to 90 min MCAO and 24 h reperfusion of the left cerebral hemisphere by the intraluminal filament method as described previously^16, 17^. In short, the left common carotid artery was exposed through a midline neck incision. Through the common carotid artery, a surgical nylon thread (Ethicon 4-0, coated with silicone) was placed in the internal carotid artery and gently advanced until a resistance was felt and a sharp decline was seen in the blood flow trace. MCAO was accepted as a decrease in laser Doppler flowmeter signals to lower than 20% of baseline. Body temperature was kept at 37 ± 0.5 °C with a heating feedback control system during surgery. At the end of 90 min of MCAO, reperfusion was initiated by withdrawing the thread, and the incisions were sutured. After the animals recovered from anesthesia, they were returned to a warm cage during the 24-h reperfusion period.

### Behavioral studies

#### Assessment of neurological outcome

Behavioral tests were accomplished by a blinded observer 24 h after surgery or MCAO in the studied groups. As described previously, a five-point neurological deficit score (NDS) test was used to estimate the neurological outcome^18^. Briefly, 1: normal motor activity, 2: flexion of contralateral forelimb upon lifting by the tail, 3: circling to the contralateral side of the brain lesion, 4: losing of the righting reflex and reduced resistance to lateral push, and 5: no spontaneous motor activity.

#### Grip strength test

The grip strength test was performed on the paretic (right) forelimb of animals before surgery and again 24 h after MCAO. A digital recording (in grams) of three consecutive trials was made for each rat and the means of three results were used for analysis. Grip strength performance was expressed as a ratio over the baseline (pre-stroke) value^19^.

#### Hot plate test

The hot plate test was used to evaluate the rats’ sensory function before surgery and 24 h after MCAO. Nociception was assessed by recording the latency time to lick a hind paw when the rat was placed on a 50 °C plate. The rat was displaced from the plate instantly upon licking the hind paw or if no reaction occurred during 50 s^20^.

#### Measurement of cerebral infarct size

Six slices (2 mm thick) were coronally dissected from brain tissue and stained with a 2% solution of 2,3,5-triphenyltetrazolium chloride (TTC) (Molekula, UK). Images of the stained sections were taken, and the infarction areas were measured by using image analyzer software (NIH Image Analyzer). Brain infarction volume was calculated as described previously^17^.

#### Brain edema detection

Absolute brain water content (BWC) as a direct index of brain edema was detected by the wet/dry weight method. Briefly, the wet weight (WW) of each hemisphere was measured and recorded. Then, the tissues were placed for 24 h in a 110 °C oven to obtain the tissue dry weight (DW), and BWC of each hemisphere (%) was determined using the following equation^21^.

### BWC (%) = [(WW − DW)/WW] × 100

#### Assessment of BBB permeability

Blood-brain barrier permeability was assessed by the Evans blue extravasation technique^16, 22^. Briefly, 2% Evans blue (EB) solution (1 ml/kg, Sigma Chemical Co., UK) was slowly injected via left jugular vein 30 min after neck surgery. At the end of the reperfusion time, the animal’s circulation was perfused by 250 ml warm normal saline (37 °C). After decapitation, each hemisphere of the brain was carefully weighed and homogenized in 2.5 ml phosphate buffered saline and then mixed with 2.5 ml trichloroacetic acid (60%). EB absorbance was measured at 610 nm, and the concentration (µg/g wet tissue) was calculated using a standard curve.

#### Assessment of brain tissue antioxidant power

Ischemic hemispheres were dissected, weighed, and stored at −80 °C until analysis. Tissues were homogenized in PBS with a weight-to-volume ratio of 1:5 and then centrifuged (20,000 g, 4 °C) for 30 min. Total antioxidant activity was detected by ferric reducing antioxidant power (FRAP) assay^23, 24^. The working FRAP reagent was prepared by mixing acetate buffer, TPTZ (2,4,6-tripyridyl-s-triazine) solution and FeCl_3_ at a ratio of 10:1:1. Ten microliters of supernatant was added to 300 μl of prepared reagent and warmed to 37 °C for 10 min. Then, the absorbance of samples was measured at 593 nm. Concentrations were calculated against a standard curve (100–1000 μM, FeSO_4_.7H2O) and data were expressed as μM per g of wet tissue.

#### Statistical analyses

The data are presented as mean ± SEM and the significance of differences between groups was estimated by Student’s *t*- test or one-way analysis of variance (ANOVA) followed by Tukey’s or Dennett’s test. Statistical significance was accepted at *P* < 0.05.

## Results

### Behavioral assessments

**Figure 1:**
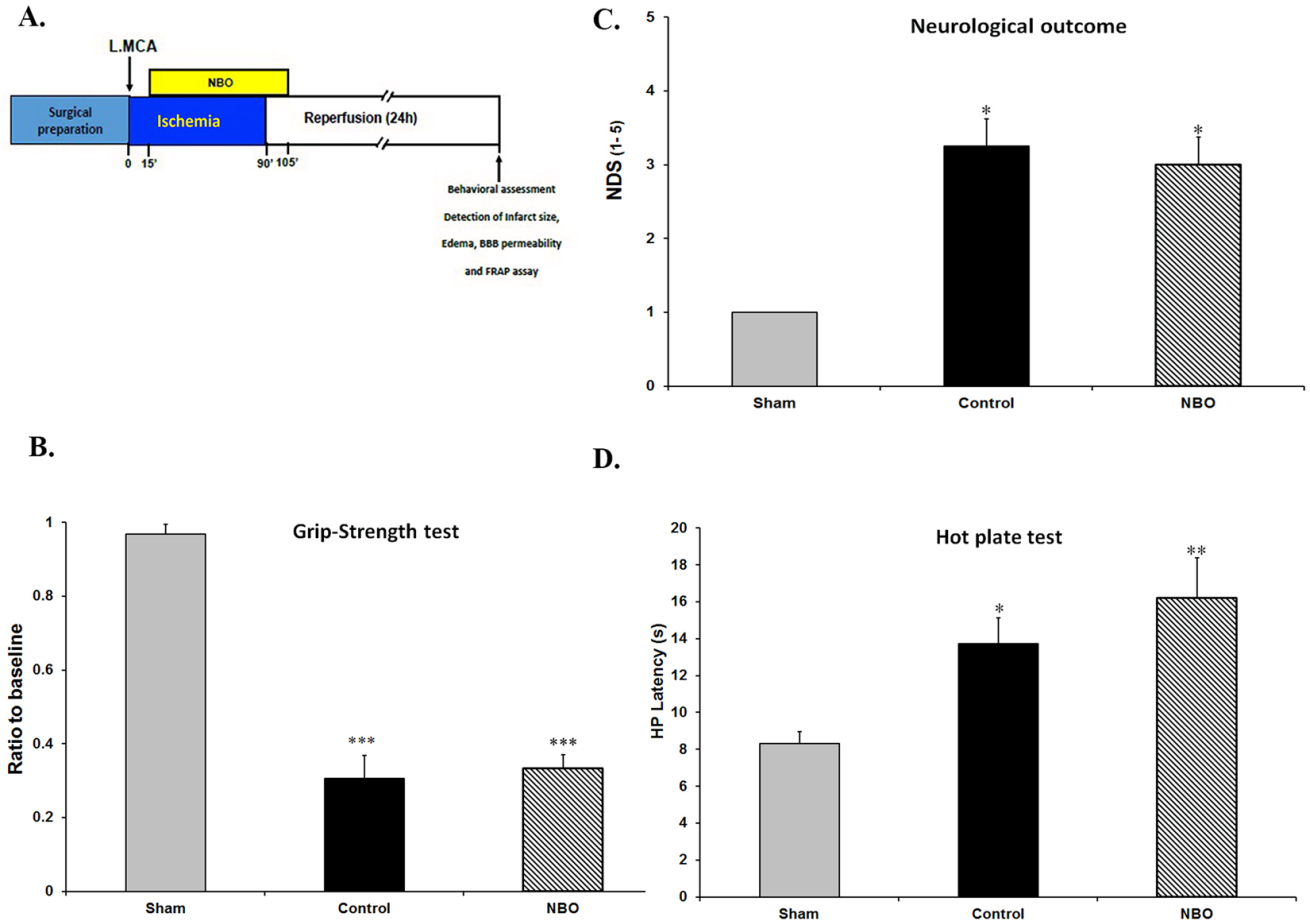
Effects of NBO treatment on sensorimotor deficits. (**A**) Schematic representation of the experimental design. (**B**) Grip strength test assessment on paretic (right) forelimb 24 h after MCAO. (**C**) Neurological deficit score (NDS) in the studied groups. (**D**) Hot plate latency (HP) results 24 h after MCAO. Values are mean ± SEM (*n* = 6–8). *P < 0.05, **P < 0.01 and ***P < 0.001 compared with sham group.

Ischemia produced severe sensory and motor disabilities in control ischemic rats in comparison with sham animals. Ischemia increased the neurological deficit score (NDS) of ischemic rats in the control group compared with sham animals (*P* < 0.001), and early oxygen therapy did not produce any improvement in neurological function (Fig. 1C).

To evaluate motor function, animals were examined by the grip strength test. When control ischemic animals had severe motor disabilities compared with the sham group (*P* < 0.001), early NBO supplementation was not associated with a significantly better performance nor motor recovery (Fig. 1B).

In addition to motor performance, the recovery of sensory function in ischemic animals was also examined by the hot plate test. Ischemia significantly enhanced foot licking time of control animals in the hot plate test compared with the sham group (*P* < 0.05). Oxygen therapy did not improve sensory function in the hot plate in comparison with the control group (Fig. 1D). These results jointly show that early oxygen therapy could not significantly produce recovery from ischemic motor and sensory deficits.

### Assessment of cerebral infarct volumes and mortality rate

**Figure 2:**
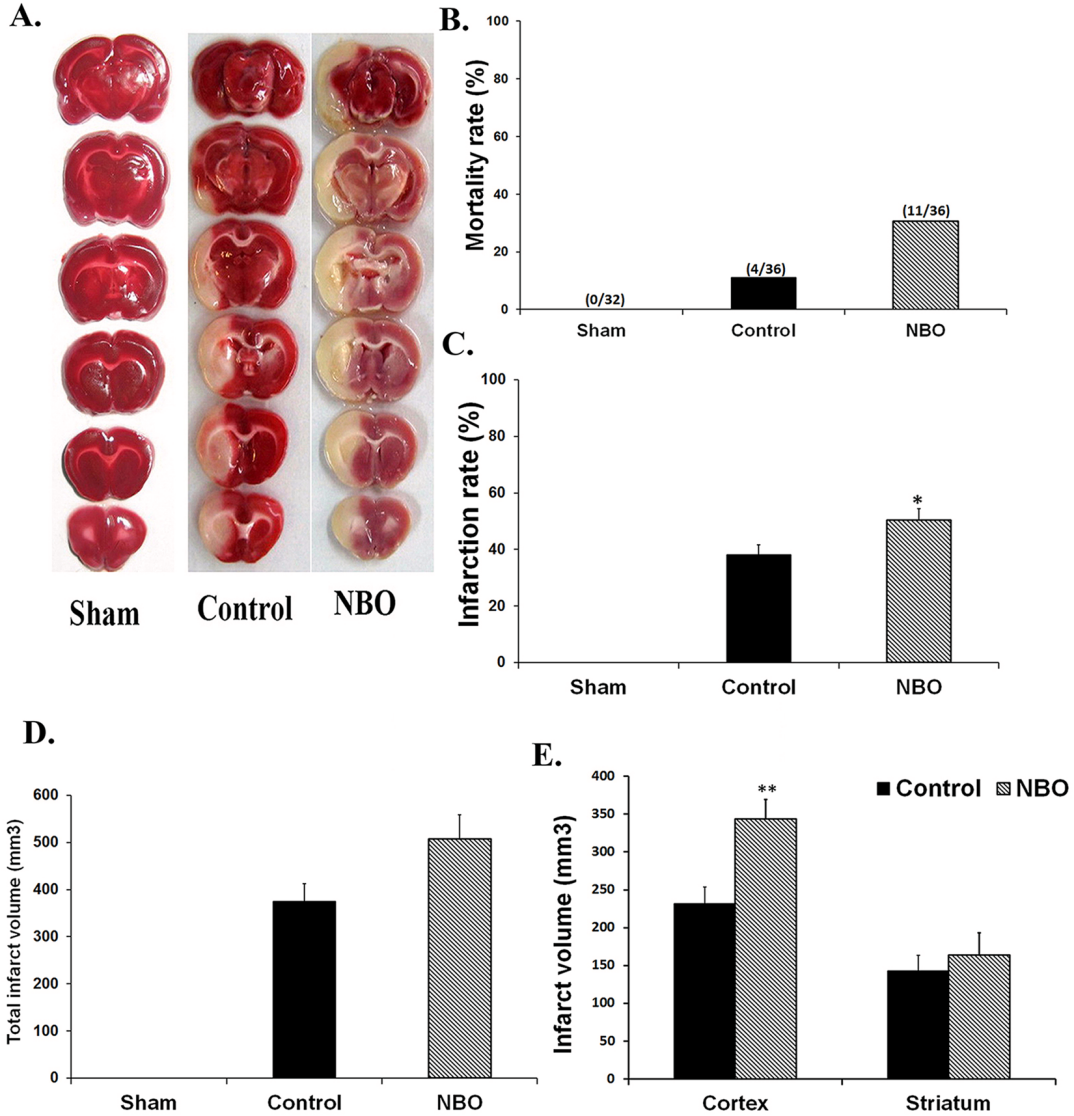
Effects of NBO treatment on ischemic brain injury. (**A**) Brain slices stained with TTC in the studied groups. Infarct region was illustrated with white color. (**B**) Mortality rate in the studied groups. (**C–E**) Infarct ratio, total, cortical, and striatal infarct sizes in the studied groups (*n* = 6–8). Values are mean ± SEM. *P < 0.05, **P < 0.01 compared to control group.

Sham-operated rats had no infarctions. Ninety minutes’ ischemia and 24 h reperfusion caused brain injury by 38% of left hemispheres in control and 50% in NBO-treated groups. The total infarct volume in control and oxygen-treated ischemic rats was not significantly different (374 ± 38 mm^3^ and 507 ± 51 mm^3^, respectively). Additionally, there was no significant difference in striatal infarction volume between control and NBO-treated groups, but cortical infarction volume and infarction rate of the lesioned hemisphere in the NBO-treated group were significantly greater than those of the control ischemic group (*P* < 0.01 and *P* < 0.05, respectively, Fig. 2A–E).

As shown in Fig. 2B, the mortality rate in the NBO-treated group (30%, 11 of 36 rats) was dramatically higher than in the control ischemic (11%, 4 of 36 rats) and sham groups (0%, zero of 32). Postmortem examination of deceased animals showed that all of them died due to severe brain infarction and edema. No subarachnoid hemorrhage was observed.

### Detection of ischemic edema formation

**Figure 3:**
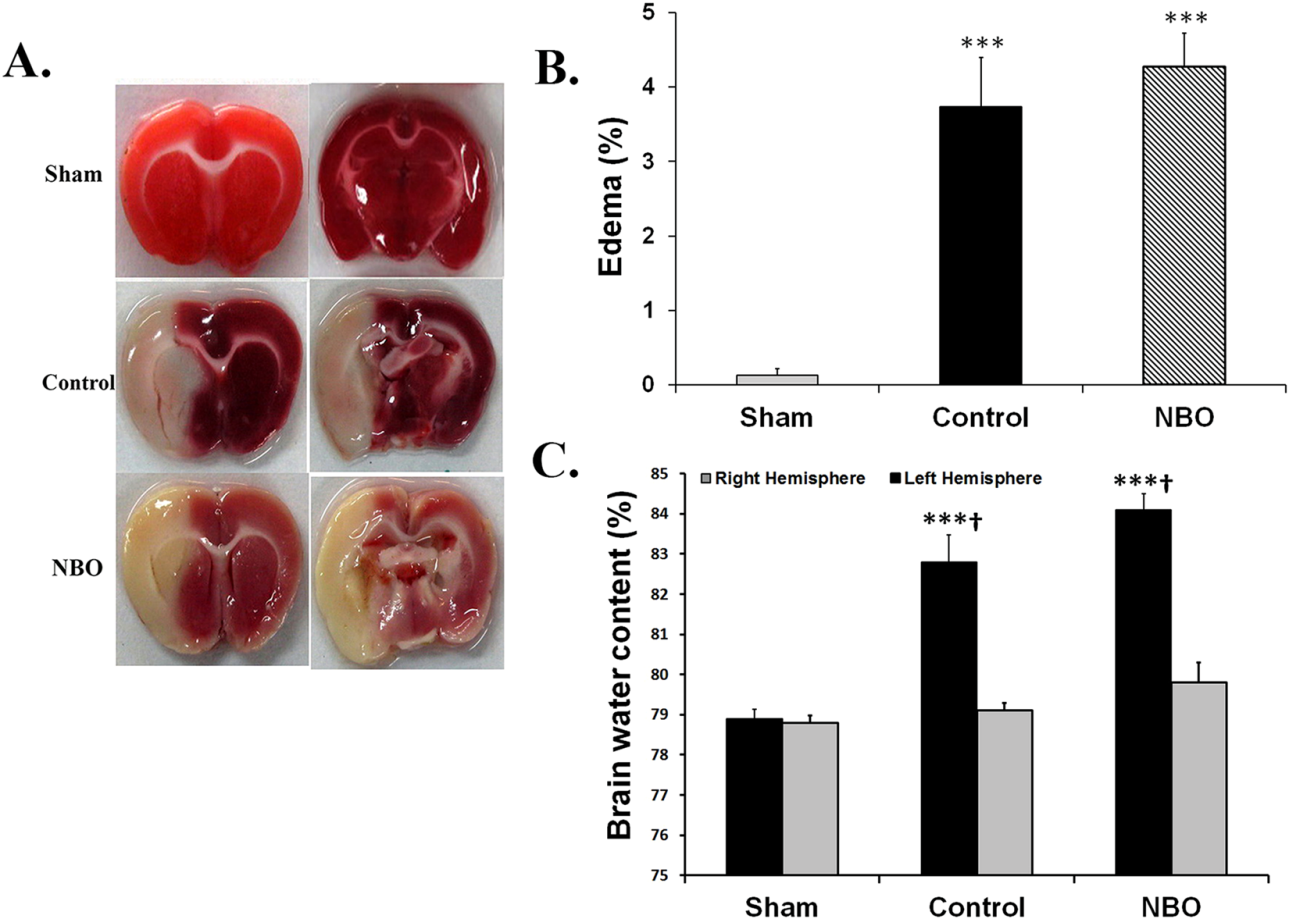
Effects of NBO treatment on ischemic brain edema formation. (**A**) Brain section stained with TTC from animals that studied in experimental groups. Sever edema caused noticeable swelling and deformation of the left hemisphere in control and NBO-treated animals. (**B**) Brain edema formation in experimental groups. (**C**) Brain water content in the left and right sides of the brains in the studied groups (n = 7–8). Values are mean ± SEM. (***P < 0.001, compared with sham group; ^**†**^ *P* < 0.001, compared with right side of the same group).

The absolute values of brain water content (BWC) of the right hemispheres of experimental groups were not statistically different. Ischemia elevated BWC of the lesioned hemispheres (left) of control-ischemia rats to 82.8 ± 0.67% compared with sham animals (78.9 ± 0.24%, *P* < 0.001). Nevertheless, early NBO supplementation did not significantly diminish BWC (84.1 ± 0.41%, Fig. 3A–C).

The brain edema of control ischemic rats (3.73 ± 0.67%) was significantly greater than that of sham-operated rats (*P* < 0.001). Early oxygen therapy did not protect against brain edema formation provoked by ischemia/reperfusion injury (4.27 ± 0.45%, Fig. 3B).

### Evaluation of BBB permeability

**Figure 4:**
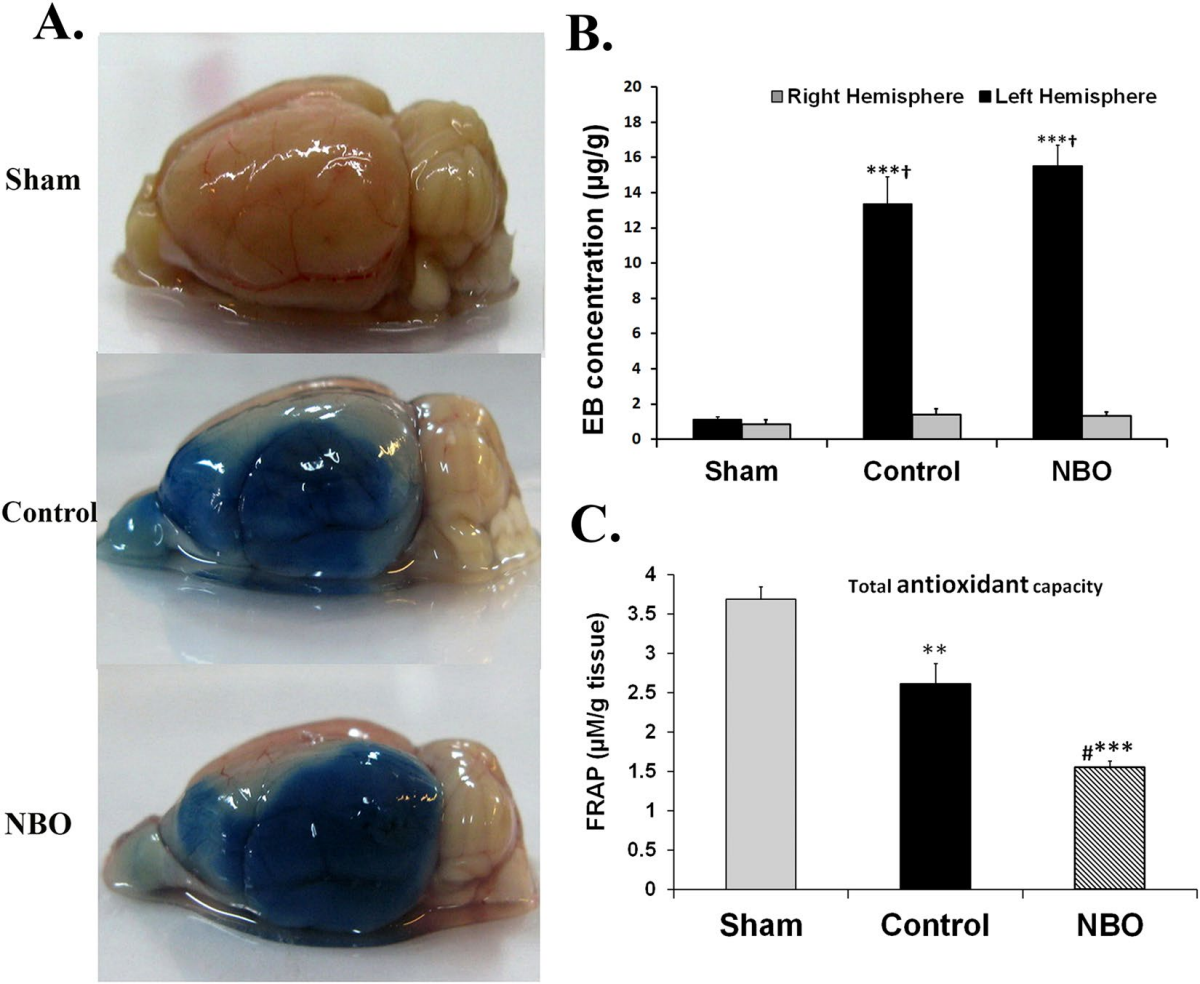
Effects of NBO treatment on ischemia-induced BBB destruction. (**A**) Photograph of brains in rats from studied groups 24 h after MCAO and Evans blue injection. The intensity of blue color depicted in control and NBO treated rats is related to the extent of damage to the cerebral vasculature of the lesioned side. (**B**) EB concentration in the left and right side of the brains in the studied groups (n = 6–7). (**C**) Assessment of brain tissue antioxidant power based on FRAP value in experimental groups (n = 6–9). Values are mean ± SEM. (***P < 0.001, **P < 0.01 compared with sham group; ^*#*^ *P* < 0.01 compared with control group; ^**†**^ *P* < 0.001 compared with right side of the same group).

There was no statistically significant difference in EB concentrations in the right brain hemispheres between experimental groups. EB concentration of the ischemic hemisphere in control (13.37 ± 1.54 µg/g) and NBO-treated rats (15.51 ± 1.17 µg/g) were significantly greater than that of sham-operated rats (1.13 ± 0.13 µg/g, *P* < 0.001). Additionally, oxygen therapy had no beneficial effects on ischemia-induced BBB integrity disruption compared with the control group (Fig. 4A,B).

### Assessment of brain tissue anti-oxidant power

Ninety-min-long ischemia and 24-h-long reperfusion in the control ischemic group resulted in significantly decreased FRAP values in the left side of the brain tissue, as compared with the sham group (*P* < 0.01). Early NBO treatments provided a significantly reduced FRAP value as compared with the control ischemic (*P* < 0.01) and sham (*P* < 0.001) groups and caused a 58% reduction in brain tissue antioxidant power. Furthermore, oxygen therapy exacerbated ischemia/reperfusion-induced oxidative stress in brain tissue (Fig. 4C).

## Discussion

The results of the present study show that early oxygen therapy following acute ischemic stroke does not reduce vasogenic brain edema, nor does it protect against oxidative stress-induced BBB destruction in adult male Sprague-Dawley rats. Additionally, cerebral edema formation occurs in conjunction with an increased mortality rate, serious brain injury, and impairment of brain antioxidant power.

Some previous studies reported that NBO reduces ischemic brain injury, improves neurological outcome, and protects the BBB against ischemic disruption, especially when used early after ischemia and for a short duration^5, 7, 14, 25^. These studies suggest that NBO may act as an effective and safe treatment strategy for ischemic stroke^14, 15^. However, our findings showed that early oxygen supplementation did not produce beneficial effects against ischemic brain damage and subsequent edema formation. Our research showed that early oxygen therapy may exacerbate oxidative cerebral damage and produce harmful effects in acute ischemic stroke. Other studies also showed that early oxygen therapy increased ischemia-induced brain damage and mortality rate after stroke^9, 10, 12^. Our results are in accordance with previous studies, which reported that reoxygenation following moderate hypoxia caused BBB impairment through ROS generation and subsequent tight junction protein destruction^26–28^.

There are multiple reasons for the failure of NBO to produce protective effects against cerebral ischemic injuries and vasogenic edema formation. Early hyperoxia after cerebral ischemia could increase the generation of ROS due to mitochondrial respiration impairment^12, 13^. Thus, NBO may cause severe oxidative damage of the brain and BBB disruption by lipid peroxidation, especially during reperfusion^6, 29^. Our findings also showed that the antioxidant power of brain tissue was significantly reduced in NBO-treated ischemic rats. In addition, hyperoxia may compromise cerebral blood flow via vasoconstriction and thereby worsen the functional outcome of ischemic stroke^13, 30^. Dubinsky *et al*. also suggested that neuronal cell death caused by glutamate excitotoxicity requires the presence of oxygen^31^.

It appears that treatment of acute ischemic stroke might depend on the severity of the brain injury, timing and duration of oxygen therapy, and the age of the patient. Oxygen therapy may exacerbate stroke outcome in aged patients with severe brain damage^13^. However, more studies should be conducted to clarify the beneficial effects and potential side effects of NBO treatment before clinical use. Further experimental studies are needed to determine the effects of different percentages of oxygen, duration of NBO supplementation, duration of ischemia, and timing of treatment. New experimental research should be designed using aged, hypertensive, and diabetic animals concerning stroke-related risk factors.

In conclusion, our findings showed that early NBO treatment did not reduce ischemic brain injuries and does not produce protective effects against vasogenic cerebral edema formation and BBB disruption following acute ischemic stroke. Reduction of brain tissue antioxidant power and increases in lipid peroxidation may be a possible mechanism for early oxygen therapy failure. Further experimental studies are needed to clarify the beneficial effects and potential side effects of early oxygen therapy before clinical use.
